# Human DNA ligases I and III have stand-alone end-joining capability, but differ in ligation efficiency and specificity

**DOI:** 10.1093/nar/gkac1263

**Published:** 2023-01-10

**Authors:** Justin R McNally, Amanda M Ames, Suzanne J Admiraal, Patrick J O’Brien

**Affiliations:** Department of Biological Chemistry, Michigan Medicine, University of Michigan, Ann Arbor, MI 48109, USA; Department of Biological Chemistry, Michigan Medicine, University of Michigan, Ann Arbor, MI 48109, USA; Department of Biological Chemistry, Michigan Medicine, University of Michigan, Ann Arbor, MI 48109, USA; Department of Biological Chemistry, Michigan Medicine, University of Michigan, Ann Arbor, MI 48109, USA

## Abstract

Double-strand DNA breaks (DSBs) are toxic to cells, and improper repair can cause chromosomal abnormalities that initiate and drive cancer progression. DNA ligases III and IV (LIG3, LIG4) have long been credited for repair of DSBs in mammals, but recent evidence suggests that DNA ligase I (LIG1) has intrinsic end-joining (EJ) activity that can compensate for their loss. To test this model, we employed *in vitro* biochemical assays to compare EJ by LIG1 and LIG3. The ligases join blunt-end and 3′-overhang-containing DNA substrates with similar catalytic efficiency, but LIG1 joins 5′-overhang-containing DNA substrates ∼20-fold less efficiently than LIG3 under optimal conditions. LIG1-catalyzed EJ is compromised at a physiological concentration of Mg^2+^, but its activity is restored by increased molecular crowding. In contrast to LIG1, LIG3 efficiently catalyzes EJ reactions at a physiological concentration of Mg^2+^ with or without molecular crowding. Under all tested conditions, LIG3 has greater affinity than LIG1 for DNA ends. Remarkably, LIG3 can ligate both strands of a DSB during a single binding encounter. The weaker DNA binding affinity of LIG1 causes significant abortive ligation that is sensitive to molecular crowding and DNA terminal structure. These results provide new insights into mechanisms of alternative nonhomologous EJ.

## INTRODUCTION

**Figure 1.**
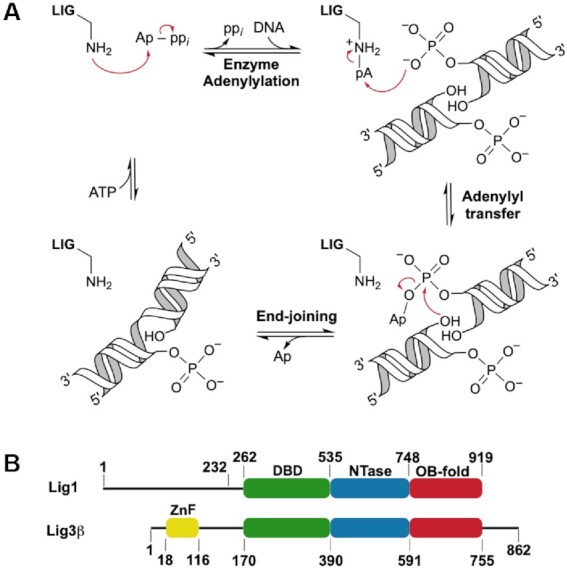
Human LIG1 and LIG3 share mechanistic and structural features. (**A**) Mechanism for blunt-end ligation by ATP-dependent DNA ligases. The end-joining reaction produces a nicked DNA product (singly ligated product) that must undergo a second round of catalysis to completely repair the DNA molecule. (**B**) LIG1 and LIG3β domains and their corresponding structural boundaries. LIG1 and LIG3β possess conserved DNA binding (DBD, *green*), nucleotidyltransferase (NTase, *blue*), and oligonucleotide-binding (OB-fold, *red*) domains. LIG3 is the only DNA ligase with a zinc finger domain (ZnF, *yellow*). LIG1 with an N-terminal truncation of 232 residues (Δ232 LIG1) was used in this study.

The human genome is continuously assaulted by endogenous and exogenous DNA damaging agents. DNA replication, repair, and recombination pathways work together to maintain and safeguard the stability of the genome from these insults. Human DNA ligases I (LIG1), III (LIG3) and IV (LIG4) catalyze the ultimate step in these pathways, the joining of DNA fragments to restore continuous chromosome strands. DNA ligases employ a three-step chemical mechanism (Figure 1A) that is amenable to joining single- or double-strand DNA breaks (SSBs and DSBs, respectively) (1–3). In the first step, ATP is used to form a high-energy lysine-AMP intermediate. This activated enzyme intermediate binds to a 5′-phosphorylated SSB or DSB and transfers AMP from the active site lysine to the 5′-phosphate to form a 5′-5′ adenylylated DNA intermediate. During SSB ligation, the 3′-hydroxyl adjacent to the adenylylated 5′-phosphate is appropriately positioned for efficient sealing of the phosphodiester DNA backbone, completing the final chemical step of ligation with the release of AMP and the repaired DNA molecule. The ligation activities of LIG1 and LIG3 for the repair of SSBs have been previously characterized (4–8). For the end-joining (EJ) of DSBs that lack cohesive ends, ligases must overcome the additional challenge of properly positioning two DNA molecules to catalyze the formation of the phosphodiester bond without the aid of intermolecular base pairing interactions (Figure 1A). Previous studies have reported on the EJ activity of human LIG3 (5,6,9–13), but we are not aware of any studies showing that purified human LIG1 can catalyze EJ. Indeed, it is generally accepted that LIG1 does not perform EJ, and that this characteristic sets it apart from LIG3 and LIG4 (2,14,15).

LIG1 and LIG3 share a high degree of structural conservation within their three-domain architecture that is comprised of a DNA binding domain (DBD), a nucleotidyltransferase domain (NTase), and an oligonucleotide-binding fold (OB-fold), but they have only 22% sequence identity across this region (Figure 1B) (4,6,16). Outside of their conserved core structures, the LIG1 and LIG3 enzymes differ completely in their N- and C-termini, and it is suggested that these flanking regions are important for directing their biological activities. LIG3 has an N-terminal zinc finger (ZnF) domain that is homologous to those of poly (ADP-ribose) polymerase 1 (PARP-1; Figure 1B) (17,18). This N-terminal region of LIG3 appears to be dynamic and has been proposed to serve as a nick sensor for SSB ligation, as well as to play an essential role in LIG3-catalyzed EJ (5,6,9,12,17–20). In contrast, the N-terminus of LIG1 (∼260 residues) does not contribute to catalysis (7). In the cell, the N-terminus of LIG1 targets the enzyme to the nucleus, facilitates protein-protein interactions, and is post-translationally modified (4,14). The differences in the N-termini of LIG1 and LIG3 have led to the consensus view that LIG3, but not LIG1, is capable of performing EJ reactions due to its ZnF, which constitutes an independent DNA binding module (20).

Two recent studies used cultured mouse B-cells to demonstrate that EJ can occur during class switch recombination (CSR) in the absence of both LIG3 and LIG4 (21,22). As CSR requires DSB ligation, these data implicate LIG1 as the source of the EJ activity. Furthermore, LIG1 has been proposed to participate in alternative nonhomologous EJ, catalyzing the formation of deleterious sister chromatid telomere fusions (23). Roughly three decades prior to these studies, two research groups reported that LIG1 homologs purified from rat liver and calf thymus showed some *in vitro* EJ activity (24,25). More recently, EJ activity in human cell lysates was attributed to both LIG1 and LIG3 (26). Together, these results suggest that LIG1 could participate in an undefined EJ pathway that overlaps with the EJ activities of LIG3 and LIG4. However, there is no mechanistic information to address how LIG1-catalyzed EJ may occur, and this activity would be at odds with the widely held view that LIG1 is specialized for replicative ligation of SSB intermediates.

We took a systematic biochemical approach to characterize the EJ activities of LIG1 with an N-terminal truncation of 232 residues (Δ232 LIG1) and LIG3β on DNA ends of varying structures. We clearly demonstrate that LIG1 can catalyze EJ, with a substrate preference for DSBs with 3′-overhangs or blunt ends over DSBs with 5′-overhangs. For all EJ substrates tested, the catalytic efficiency of LIG1 decreases and its propensity to accumulate dead-end AMP-DNA species increases at a physiological concentration of Mg^2+^. In contrast, LIG3 efficiently joined all tested DSB substrates, with a modest preference for overhangs, and its catalytic efficiency was similar at both saturating and physiological Mg^2+^ levels. To simulate the crowded conditions within the cell, we used polyethylene glycol as an inert molecular crowding agent, which increased the catalytic efficiency of LIG1 by up to 100-fold but did not further improve the efficiency of LIG3. Strikingly, molecular crowding increased the lifetime of the LIG3•DNA complex, enabling LIG3 to perform the sequential ligation events required to completely repair both strands of a DSB in a single DNA binding encounter. This work highlights similarities and differences between LIG1- and LIG3-catalyzed EJ and documents, for the first time, their catalytic efficiencies for *in vitro* EJ.

## MATERIALS AND METHODS

### Recombinant LIG1 and LIG3β

Human DNA LIG1 N-terminal truncation mutant Δ232 was expressed from a pET19 vector containing an N-terminal His_6_-PreScission protease cleavable tag in Rosetta2 (DE3) *Escherichia coli* by auto induction in terrific broth and purified using a three-step procedure described previously (27). Nuclear LIG3β was expressed from a modified pET28 vector containing an N-terminal His_6_-SUMO protease cleavable tag in C41 pRare2 (DE3) *E. coli* by IPTG induction in Luria broth and purified using a four-step procedure described previously (8). An SDS-polyacrylamide gel of the purified proteins used in this study is shown in Supplementary Figure S1.

### DNA oligonucleotides

Oligonucleotides were purchased from Keck Oligonucleotide Synthesis facility and Integrated DNA Technologies, and DNA sequences are shown in Supplementary Table S1. The two SLPBE oligonucleotides were purified by ion-exchange HPLC, and the remaining oligonucleotides were purified using DNA denaturing gels (15% (w/v) polyacrylamide, 8 M urea, 1 × TBE) (7). The 28mer SSB DNA substrate that was used to determine active LIG1 and LIG3β concentrations has been described previously (7,8). The EJ substrates were 5′-phosphorylated, and their concentrations were determined using the absorbance of their internal dT-fluorescein fluorophore at 495 nm. EJ substrates and the synthetic versions of singly ligated products were annealed in annealing buffer (10 mM NaMES, pH 6.5, 50 mM NaCl) by heating the solution to 95°C for 5 min and then cooling to 4°C at a rate of 12°C/min. DNA concentrations described in assays refer to the amount of product capable of forming (e.g. 100 nM blunt-end substrate consists of 200 nM of ligatable hairpin ends).

### Gel-based EJ assay

Ligation reactions were performed at 37°C in standard reaction buffer (50 mM NaMOPS, pH 7.5, 10% glycerol, 1 mM ATP, 1 mM DTT, 100 μg/μl BSA) at 150 mM ionic strength, unless otherwise stated. Ionic strength was controlled using the Debye–Hückel theory of electrolytes. Mg(OAc)_2_ and NaOAc concentrations were adjusted to achieve the desired reaction conditions. Reactions were stopped by mixing reaction aliquots with an appropriate volume of 1.2× quench solution (90% formamide, 50 mM EDTA, 0.006% bromophenol blue, 0.006% xylene cyanol) at predetermined times. Quenched samples were loaded directly onto running DNA denaturing gels (15% (w/v) polyacrylamide, 8 M urea, 1 × TBE). Gels were scanned using a Typhoon imaging system (GE) set to monitor emission at 525 (BP20) with excitation set to 488 nm. The fluorescence values of all DNA species were within the linear range of the instrument and were quantified using ImageQuant TL (GE). After quantification, the fractions of all observable species were plotted and analyzed using GraphPad Prism.

### Multiple-turnover EJ by LIG1 and LIG3β

Initial rates of EJ reactions were determined by linear regression of the formation of ≤10% product. Product was defined as the sum of singly and doubly ligated DNA species. LIG1-catalyzed EJ efficiencies were determined by mixing 5 nM LIG1 with 25, 50, 100 and 200 nM DNA substrate at 2 and 20 mM Mg(OAc)_2_. Free Mg^2+^ concentrations of 1 and 18 mM were estimated for the reactions containing 2 and 20 mM Mg^2+^, respectively, based on the *K*_d_ values of the ATP•Mg^2+^ (12 μM) and ATP•2Mg^2+^ (17 mM) complexes, as described previously (8). Catalytic efficiencies (*k*_cat_/*K*_M, DNA_) for LIG1 were determined by linear regression of the dependence of the initial reaction rates (*V*_0_/[E]) on the concentration of each DNA substrate. LIG3β-catalyzed EJ efficiencies were determined by mixing 1 nM LIG3β with 5, 10, 20 and 40 nM DNA substrate at 2 and 20 mM Mg(OAc)_2_. When the initial reaction rates were linearly dependent on the concentration of DNA substrate, k_cat_/K_M, DNA_ values for LIG3β were determined by linear regression as described above for LIG1. The dependence of *V*_0_/[E] on the concentration of 5′-DSB substrate was fit using the Michaelis-Menten equation (equation 1) to determine *k*_cat_ and *K*_M, DNA_ values for the reaction of LIG3β with this substrate at 2 mM Mg(OAc)_2_. The *k*_cat_/*K*_M, DNA_ value for the reaction of LIG3β with 3′-DSB substrate at 2 mM Mg(OAc)_2_ could not be determined directly, so direct competition reactions between this substrate and the blunt-end substrate were carried out to determine its relative *k*_cat_/*K*_M, DNA_ value (equation 2). Competition experiments contained 4 nM LIG3β, 30 nM 3′-DSB substrate, and 200 or 400 nM blunt-end substrate, in the presence of 2 mM Mg(OAc)_2_. The Mg^2+^ concentration dependences for blunt-end ligation (0–30 mM Mg(OAc)_2_) were determined in standard reaction buffer containing 5 nM of LIG1 or LIG3β and 300 nM blunt-end substrate. Plots of initial rates as a function of free Mg^2+^ concentration were fit to a two-metal random binding model for LIG1 (equation 3) and a one-metal binding model for LIG3β (equation 4). The fraction of abortive ligation events was calculated from the concentration of adenylylated DNA intermediate (I) and ligation product (P) formed at various times during the initial rates portion of EJ reactions (equation 5). Reactions in the presence of 0–20% (w/v) polyethylene glycol 6000 (PEG) contained 5 nM LIG1 or LIG3β, 200 nM blunt-end substrate, and 2 mM Mg(OAc)_2_ in standard reaction buffer.

(1) \begin{eqnarray*} {\rm{\ }}\frac{{{{\boldsymbol{V}}}_0}}{{\left[ {\boldsymbol{E}} \right]}} = {\boldsymbol{\ }}\frac{{{{\boldsymbol{k}}}_{{\boldsymbol{cat}}} \times \left[ {\boldsymbol{S}} \right]}}{{\left( {{{\boldsymbol{K}}}_{\boldsymbol{M}} + \left[ {\boldsymbol{S}} \right]} \right)}} \end{eqnarray*}

(2) \begin{eqnarray*} {\rm{\ }}\frac{{{{\boldsymbol{V}}}_{\boldsymbol{A}}}}{{{{\boldsymbol{V}}}_{\boldsymbol{B}}}} = \frac{\left ( ^{k_{cat}}/_{K_{M}} \right )^{A} \times [A]}{\left ( ^{k_{cat}}/_{K_{M}} \right )^{B} \times [B]} \end{eqnarray*}

(3) \begin{eqnarray*} {\rm{\ }}\frac{{{{\boldsymbol{V}}}_0}}{{\left[ {{\bf E}} \right]}} = {\boldsymbol{\ }}\frac{{{{{^{{\boldsymbol{V}}_\boldsymbol{max}}}}/ \!{_{\left[ {\boldsymbol{E}} \right]}}}}}{{\frac{{{{\boldsymbol{K}}}_1{\times}{{\boldsymbol{K}}}_2}}{{{{[{\boldsymbol{M}}{{\boldsymbol{g}}}^{2 + }]}}^2}} + \frac{{{{\boldsymbol{K}}}_1 + {{\boldsymbol{K}}}_2}}{{[{\boldsymbol{M}}{{\boldsymbol{g}}}^{2 + }]}} + 1}} \end{eqnarray*}

(4) \begin{eqnarray*} {\rm{\ }}\frac{{{{\boldsymbol{V}}}_0}}{{\left[ {\boldsymbol{E}} \right]}} = {\boldsymbol{\ }}\frac{{{{\boldsymbol{k}}}_{{\boldsymbol{cat}}}{\boldsymbol{\ }} \times {\boldsymbol{\ }}\left[ {{\boldsymbol{M}}{{\boldsymbol{g}}}^{2 + }} \right]}}{{\left( {{{\boldsymbol{K}}}_{{\boldsymbol{Mg}}}{\boldsymbol{\ }} + {\boldsymbol{\ }}\left[ {{\boldsymbol{M}}{{\boldsymbol{g}}}^{2 + }} \right]} \right)}} \end{eqnarray*}

(5) \begin{eqnarray*} {\boldsymbol{Fraction\ abortive\ ligation\ }} = {\boldsymbol{\ }}\frac{{\left[ {\boldsymbol{I}} \right]}}{{\left( {\left[ {\boldsymbol{I}} \right] + \left[ {\boldsymbol{P}} \right]} \right)}} \end{eqnarray*}

### Pre-steady-state analysis of EJ by LIG3β

Burst and pre-steady-state kinetic analyses of LIG3β were performed in standard reaction buffer containing 10% (w/v) PEG with 2 mM Mg(OAc)_2_. Burst experiments contained 1 and 2 nM LIG3β with 20 and 40 nM DNA substrates, respectively. Reported bursts are the sum of the singly and doubly ligated products. Time courses fit well to Equation (6) where *A*_0_ is the burst amplitude, *k* is the pre-steady-state rate constant, *V_ss_* is the steady-state velocity and *t* is time. Pre-steady-state reactions contained 20 nM LIG3β and 40 nM DNA substrate. Singly and doubly ligated products were plotted individually and observed rate constants (*k*_obs_) were determined using a two step-irreversible mechanism in the kinetics simulation program Berkeley Madonna (7).

(6) $$\begin{equation*}{\rm {\ }}\left[ {\boldsymbol{P}} \right] = {\boldsymbol{\ }}{{\boldsymbol{A}}}_0\left( {1 - {\boldsymbol{ex}}{{\boldsymbol{p}}}^{ - {\boldsymbol{k}} \times {\boldsymbol{t}}}} \right) + {\boldsymbol{\ }}\left( {{{\boldsymbol{V}}}_{{\boldsymbol{ss}}} \times {\boldsymbol{t\ }}} \right)\end{equation*}$$

### Pulse-chase experiment

Reactions were initiated by mixing enzyme and labeled substrate solutions in standard reaction buffer containing 10% (w/v) PEG with 2 mM Mg(OAc)_2_, for an initial pre-steady-state reaction containing 20 nM LIG3β and 40 nM blunt-end substrate. After a 15 s aging time the reactions were either quenched with 1.2 × quench solution or chased with unlabeled SLPBE (a synthetic version of SLP, the singly ligated product of the blunt-end substrate; Supplementary Table S1). Final chase reactions contained 11 nM LIG3β, 23 nM blunt-end substrate and LIG3β-catalyzed derivatives, and 860 nM SLPBE; samples were taken over the next 345 s and quenched with 1.2× quench solution. Control reactions in which the order of addition of chase and substrate was reversed were initiated by mixing enzyme and SLPBE, aging for 15 s, and then chasing with a mixture that approximated the concentrations of blunt-end substrate and SLP (as SLPBE-F; Supplementary Table S1) that were present at 15 s in the chase reaction. The final control reactions contained 11 nM LIG3β, 23 nM blunt-end substrate, 5.7 nM SLPBE-F and 860 nM SLPBE. No turnover of the blunt-end substrate was observed, indicating that the SLPBE chase was effective at preventing DSB ligation. However, a slow steady-state ligation of SLPBE-F was observed, consistent with multiple-turnover ligation of the pool of labeled and unlabeled SLPBE. Therefore, the true SLP_C_ and the sum DLP_Q_ + DLP_C_ in the chase reaction were determined by plotting the total product fractions of SLP and DLP against chase time and extrapolating to zero chase time by using linear regression. DLP_C_ was calculated by subtraction of the fraction of DLP present at 15 s (DLP_Q_), which was determined from the reactions quenched immediately after the 15 s aging period (nine replicates). The efficiency of sequential ligation of the blunt-end substrate by LIG3β under these conditions was calculated using equation 7:

(7) $$\begin{equation*}{\boldsymbol{Efficiency\ }} = {\boldsymbol{\ }}\frac{{{\boldsymbol{DL}}{{\boldsymbol{P}}}_{\boldsymbol{C}}}}{{\left( {{\boldsymbol{DL}}{{\boldsymbol{P}}}_{\boldsymbol{C}} + {\boldsymbol{SL}}{{\boldsymbol{P}}}_{\boldsymbol{C}}} \right)}}\end{equation*}$$

## RESULTS AND DISCUSSION

### Catalytic efficiencies of EJ by LIG1 and LIG3

**Figure 2.**
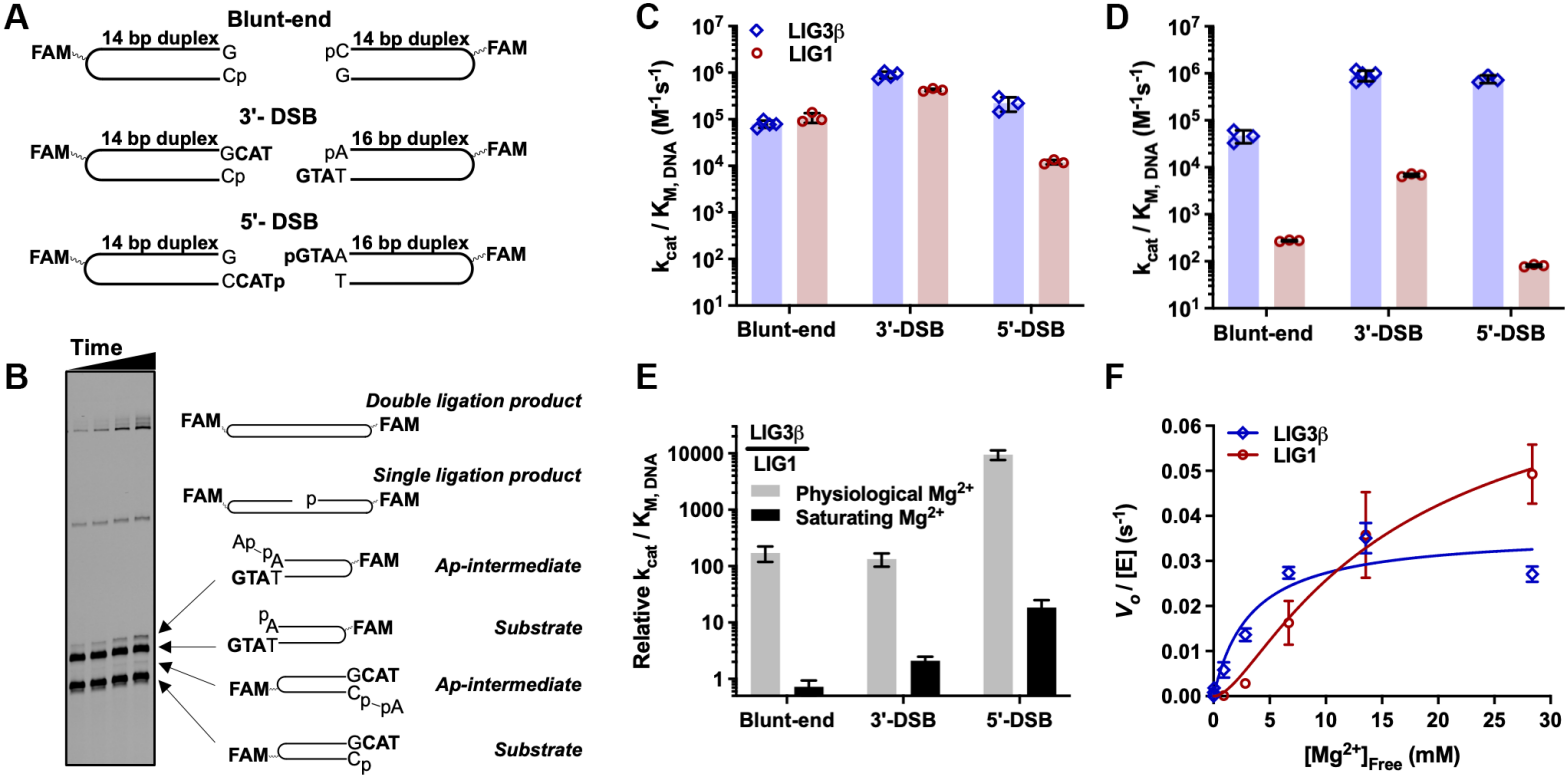
End-joining efficiencies of LIG1 and LIG3β. (**A**) Schematic of hairpin substrates used to investigate the EJ activity of LIG1 and LIG3β. Complete oligonucleotide DNA sequences are shown in Supplementary Table S1. (**B**) Representative DNA denaturing gel illustrating the band pattern of substrates, adenylylated (Ap-) DNA intermediates, singly and doubly ligated products for a LIG1-catalyzed reaction of the 3′-DSB. (**C**) Catalytic efficiency of EJ by LIG1 (red) and LIG3β (blue) at saturating Mg^2+^ concentration (18 mM free Mg^2+^). (**D**) Catalytic efficiency of EJ by LIG1 (red) and LIG3β (blue) at physiological Mg^2+^ concentration (1 mM free Mg^2+^). (**E**) Relative catalytic efficiency for LIG1 and LIG3β, expressed as LIG3β/LIG1. (**F**) Mg^2+^ concentration dependence for EJ of blunt-end DNA by LIG1(red) and LIG3β (blue). All experiments were done in at least triplicate (mean ± S.D.).

EJ reactions can be complex if there are multiple ligatable DNA ends, so we developed a simplified EJ assay featuring defined compatible ends to investigate the activity and specificity of Δ232 LIG1 and LIG3β. Fluorescently labeled DNA hairpin substrates, containing GTA trinucleotide loops closed by 14–16 base pairs (bp) of duplex DNA, were designed to have blunt ends, short 3′-overhangs (3′-DSB), or short 5′-overhangs (5′-DSB) (Figure 2A). The paired overhangs of the 3′-DSB and 5′-DSB substrates contained three nucleotides of complementary sequence. The hairpin substrates, adenylylated DNA intermediates, and singly and doubly ligated products could be separated by denaturing polyacrylamide gel electrophoresis (Figure 2B), and reaction species were verified by native polyacrylamide gel electrophoresis, comparison with synthetic standards, and exonuclease digestion (Supplementary Figure S2), as well as by observing the reaction progress associated with the formation and decay of the singly ligated product as a function of time under pre-steady-state conditions (Supplementary Figure S3). Hairpin substrates closed by 14 bp of duplex DNA were found to be of sufficient length, because blunt-end substrates with 14 and 18 bp duplexes were ligated with similar rates by both LIG1 and LIG3 (Supplementary Figure S4). These data are consistent with a footprint of roughly 8 bp on either side of a SSB that can be inferred from crystal structures of LIG1 and LIG3 with nicked DNA (4,6).

To determine the catalytic efficiencies (*k*_cat_/*K*_M, DNA_) of EJ catalyzed by LIG1 and LIG3, the dependence of reaction rate on the concentration of blunt-end, 3′-DSB, and 5′-DSB substrates was measured for each ligase in the presence of saturating Mg^2+^ (20 mM). Initial rates of EJ were linearly dependent on substrate concentration for both ligases for all substrates (Supplementary Figure S5), indicating that the tested substrate concentrations were subsaturating ([DNA] < *K*_M, DNA_). The catalytic efficiencies that were determined from these data reveal that LIG1 and LIG3 have remarkably similar *k*_cat_/*K*_M, DNA_ values for the blunt-end and 3′-DSB substrates when the Mg^2+^ cofactor is saturating, whereas the efficiency of LIG1-catalyzed EJ of 5′-DSB is ∼20-fold lower than that of LIG3 (Figure 2C). These results clearly demonstrate that LIG1 is adept at catalyzing EJ, in addition to its established function of ligating SSBs.

Cellular concentrations of free Mg^2+^ are typically 0.4–1.2 mM, so we next measured rates of DSB ligation in the presence of 1 mM free Mg^2+^ (Supplementary Figure S6). The catalytic efficiencies of LIG1-catalyzed EJ were reduced by ∼2 orders of magnitude for all three substrates at this physiological Mg^2+^ concentration (Figure 2C and D). The large effect caused by decreased Mg^2+^ is unexpected, because in SSB ligation LIG1 binds Mg^2+^ with a *K*_d, Mg_ value of 0.7 mM, which is a 10-fold higher affinity than the corresponding Mg^2+^ affinity of LIG3 (8). Although initial rates of EJ catalyzed by LIG3 at physiological Mg^2+^ remained linearly dependent on substrate concentration for the blunt-end DSB, the LIG3 reaction reached saturation at low concentrations of the 3′-DSB and 5′-DSB substrates (Supplementary Figure S6A). Catalytic efficiency values could nevertheless be determined by fitting rate data to a Michaelis-Menten saturation curve (5′-DSB; Supplementary Figure S6A) or by direct competition experiments (3′-DSB; Supplementary Figure S6B). At the physiological Mg^2+^ concentration, LIG3 had a reduced maximal turnover number (*k*_cat_) of ∼0.01 s^−1^ for both the 3′- and 5′-DSB substrates (Supplementary Figure S6A). This indicates that the availability of the Mg^2+^ cofactor limits the rate of cohesive-end ligation by LIG3 under physiological conditions, such that maximal EJ activity can be significantly enhanced by increasing the Mg^2+^ concentration. Remarkably, the catalytic efficiency (*k*_cat_/*K*_M, DNA_) of LIG3 for DSB substrates was not altered by the limiting amount of Mg^2+^ (Figure 2C and D). In other words, LIG3 remains highly efficient at capturing DSB substrates despite its weak affinity for Mg^2+^.

The striking differences in the relative catalytic efficiencies of LIG1- and LIG3-catalyzed EJ at physiological and saturating Mg^2+^ (Figure 2E) can be explained by large differences in their Mg^2+^ concentration dependences for EJ. The hyperbolic Mg^2+^ dependence of LIG3 contrasts with the sigmoidal dependence of LIG1 (Figure 2F) and accounts for why LIG3 has 100–10 000-fold greater catalytic efficiencies than LIG1 at physiological Mg^2+^ concentrations, even though the two ligases have similar catalytic efficiencies at saturating Mg^2+^ (Figure 2E). The reduced specificity of LIG1 for the 5′-DSB substrate at both Mg^2+^ concentrations may be due to the absence of a template strand for the 5′-overhang, but this absence appears not to hinder LIG3, which ligates the 5′-DSB efficiently (Figure 2C and D). In summary, these data indicate that LIG1 and LIG3 are both capable of performing EJ reactions, albeit with different preferences for DSB termini, and the catalytic efficiency of LIG1, but not LIG3, is highly sensitive to the concentration of Mg^2+^ cofactor. These distinctive biochemical features of DSB ligation could not have been anticipated based on previous characterization of SSB ligation by LIG1 and LIG3.

### Abortive ligation by LIG1

**Figure 3.**
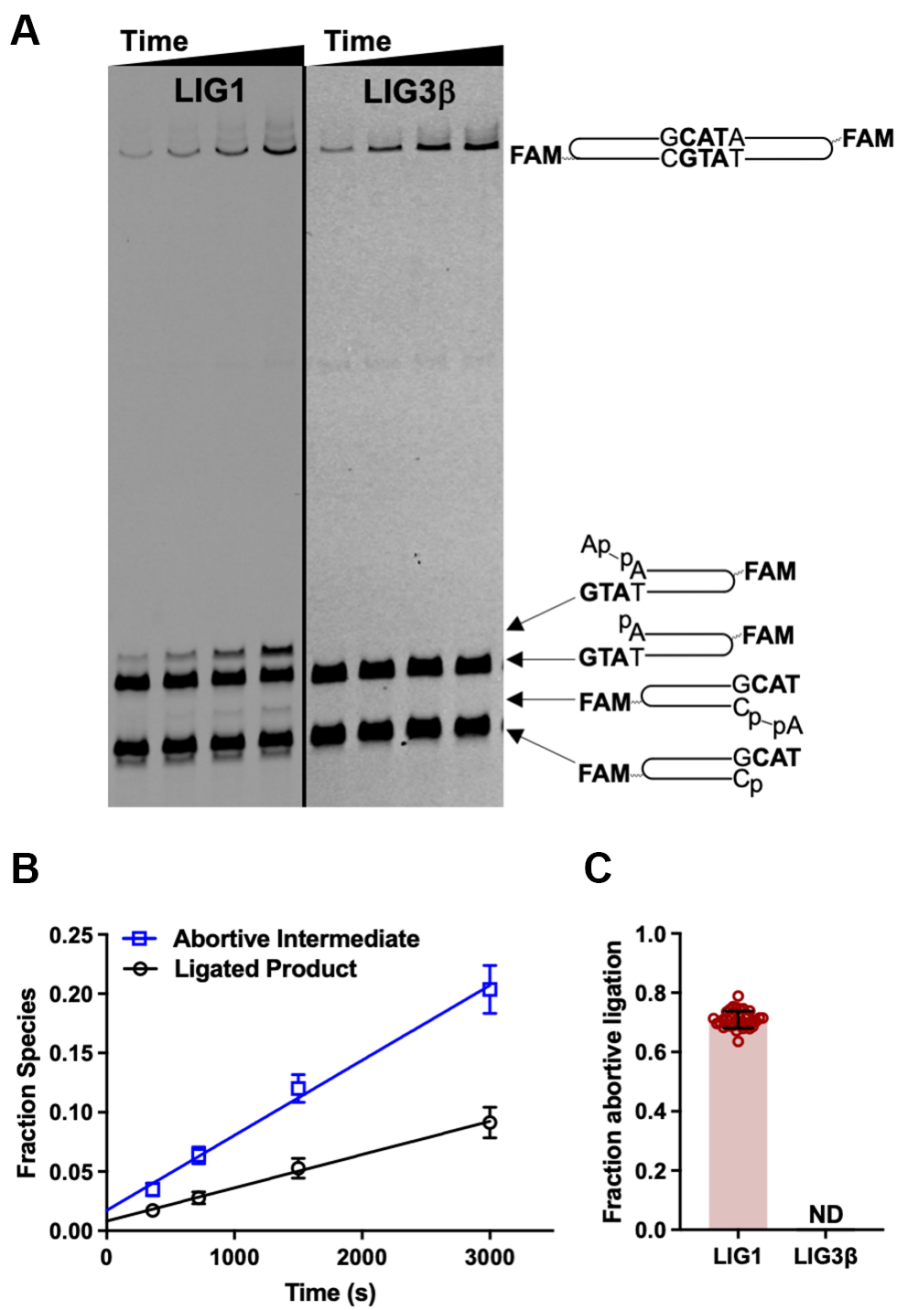
Abortive ligation by LIG1. (**A**) DNA denaturing gels comparing ligation of the 3′-DSB substrate by LIG1 and LIG3β at physiological Mg^2+^ (1 mM free Mg^2+^). (**B**) Fraction abortive intermediate and ligated product generated as a function of time by LIG1. Fraction species includes the reaction shown in **A** (left panel) and replicate reactions, all containing 100 nM 3′-DSB. (**C**) Greater than 70% of all adenylyl transfer events catalyzed by LIG1 generate adenylylated DNA intermediates, but abortive ligation by LIG3β was not detected (ND) (e.g. A, right panel). Equation 5 was used to calculate fraction abortive ligation from data obtained in reactions containing 50, 100 and 200 nM 3′-DSB (Supplementary Figure S8). Data in (B) are the average of three independent experiments, and data in (C) are the average of nine independent experiments (mean ± S.D.).

Previous studies using SSB substrates showed that LIG1 is prone to abortive ligation—premature release of the adenylylated DNA intermediate—when Mg^2+^ is limiting (7,8). In our EJ reactions with DSB substrates we also observed the accumulation of abortive species generated by LIG1, but not by LIG3, at physiological Mg^2+^ (Figure 3). LIG1 showed distinct substrate preferences, aborting ligation of the 5′-DSB substrate to a greater extent than the 3′-DSB or blunt-end substrates (Supplementary Figure S7). These observations indicate that although LIG1 can readily adenylylate a 5′-phosphorylated DNA end to form an AMP-DNA intermediate, LIG1 often releases it and rapidly reacts with a fresh molecule of ATP, resulting in the accumulation of free AMP-DNA as a dead-end species. For the 3′-DSB and 5′-DSB substrates we noticed that LIG1 abortive species were generated almost exclusively on oligonucleotides with a terminal A•T bp (Figure 3A and Supplementary Figure S7A). This result suggests that the reduced stability of a terminal A•T bp destabilizes the LIG1•AMP-DNA species, making formation of a productive EJ complex less favorable. The propensity of LIG1 to accumulate abortive intermediates was not dependent on substrate concentration: roughly 70% of all adenylyl transfer events failed to form productive EJ complexes with the 3′-DSB substrate (Figure 3C). Similarly, abortive ligation occurred at levels of 75% and 90% for the blunt-end and 5′-DSB substrates, respectively (Supplementary Figure S7B). In contrast, LIG3 did not accumulate appreciable abortive species during reaction with the DSB substrates at either physiological or saturating Mg^2+^ concentrations. Abortive ligation by LIG1 was completely alleviated at saturating Mg^2+^ concentration, which parallels observations for the Mg^2+^ dependence of abortive ligation in SSB ligation (7).

### Influence of molecular crowding on EJ activity

**Figure 4.**
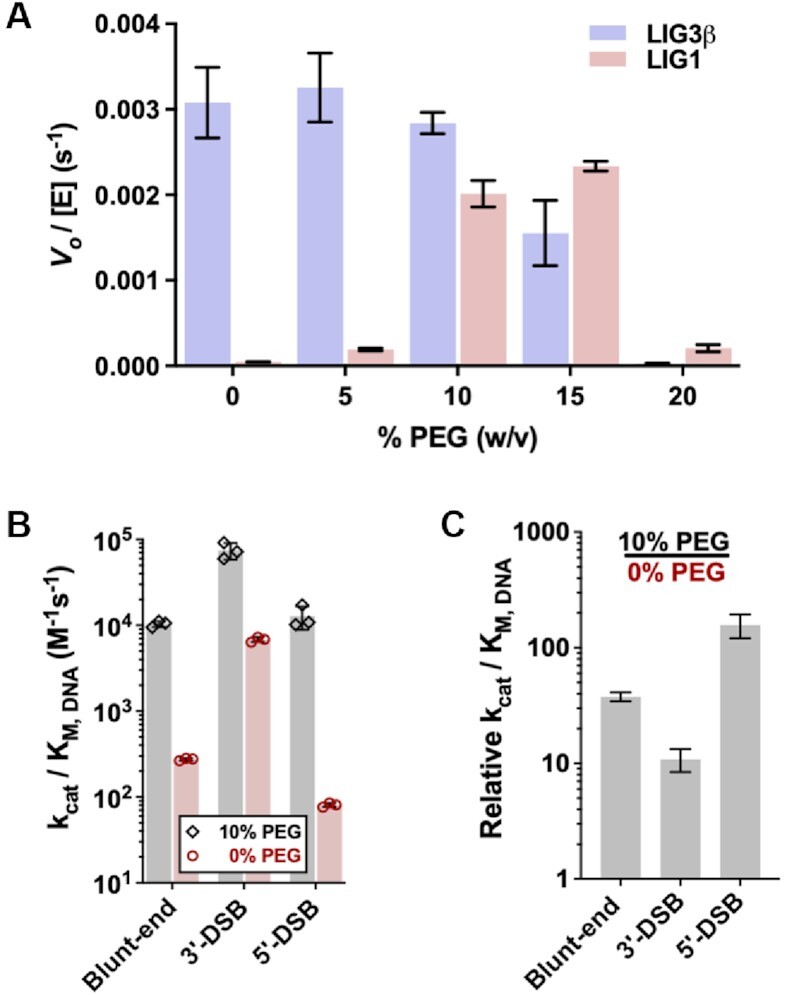
Influence of molecular crowding on end-joining activity. (**A**) Blunt-end DNA ligation by LIG1 and LIG3β in the presence of 0–20% (w/v) PEG. (**B**) Catalytic efficiency of LIG1-catalyzed EJ in the presence (gray) and absence (red) of 10% PEG. The *k*_cat_/*K*_M, DNA_ values for LIG1 reactions in the presence of 10% PEG were determined from the linear dependencies of initial rates on substrate concentrations (Supplementary Figure S9). (**C**) Relative catalytic efficiency of LIG1 in the presence and absence of 10% PEG. All reactions contained 1 mM free Mg^2+^ and were done in at least triplicate (mean ± S.D.).

We next considered the impact that the crowded cellular environment would have on the DNA EJ activity of the DNA ligases. Macromolecules occupy 20–30% of the intracellular volume, and the sum of protein and oligonucleotide concentrations is approximately 300 mg/ml (28). Molecular crowding agents influence enzymatic reactions by reducing translational diffusion, thereby aiding in substrate capture and stabilization of E•S complexes (29). To mimic the crowded environment of the cell, we employed the inert molecular crowding agent polyethylene glycol 6000 (PEG). Molecular crowding agents like PEG have been observed to increase the EJ activities of DNA ligases (13,25,30,31). A survey of ligase activity over a range of PEG concentrations indicated that concentrations greater than 10% (w/v) inhibited LIG3, whereas LIG1 activity was maximally stimulated with 10–15% PEG (Figure 4A). Addition of 10% PEG to the physiological Mg^2+^ condition increased the *k*_cat_/*K*_M, DNA_ values of LIG1-catalyzed EJ by 10- and 30-fold for the 3′-DSB and blunt-end substrates, respectively (Figure 4B and C). Strikingly, the catalytic efficiency of LIG1-catalyzed EJ of the 5′-DSB substrate increased by two orders of magnitude in the presence of 10% PEG. Furthermore, molecular crowding reduced the overall abortive ligation of each DSB substrate by 20–30% (Supplementary Figure S7B). Molecular crowding enhances the catalytic efficiency of LIG1 for ligation of DSBs, suggesting that LIG1 is capable of catalyzing such reactions within the crowded environment of a cell.

### Pre-steady-state analysis of LIG3-catalyzed EJ reveals sequential ligation events during a single DNA binding encounter

**Figure 5.**
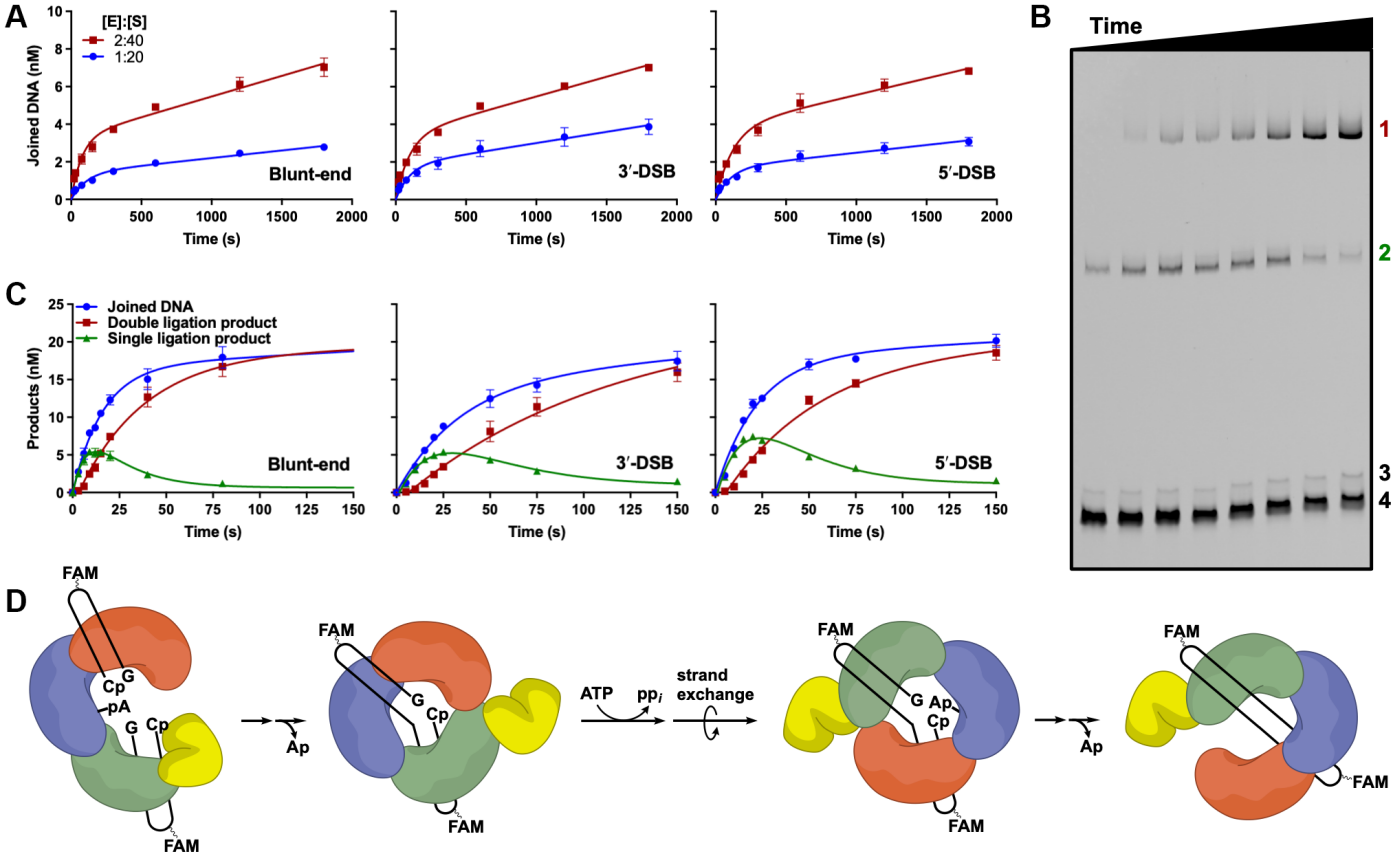
Pre-steady-state analysis of LIG3β-catalyzed EJ in 10% (w/v) PEG. (**A**) Burst analysis of 1 and 2 nM LIG3β with 20 and 40 nM EJ substrates, respectively. Joined DNA represents the sum of both singly and doubly ligated products. (**B**) Representative DNA denaturing gel illustrating the formation and decay of the singly ligated product (labeled *2*) followed by the formation of the doubly ligated product (labeled *1*) during pre-steady-state blunt-end DNA ligation. Adenylylated DNA intermediate and unreacted blunt-end substrate are labeled *3* and 4, respectively. (**C**) Pre-steady-state quantification of singly and doubly ligated products for reactions of 20 nM LIG3β with 40 nM EJ substrates. Sum of singly and doubly ligated products is shown in blue. (**D**) Simplified mechanism for sequential ligation events by LIG3β. Experiments in (A) and (C) were performed in triplicate and data are presented as mean ± S.D.

Although molecular crowding did not increase the rate of DSB ligation by LIG3 (Figure 4A), we found that it nevertheless had a substantial impact by reducing the rate of product release. In the presence of 10% PEG, LIG3 exhibits a pre-steady-state burst, during which time both strands are ligated, followed by a slower rate of steady-state ligation (Figure 5A). Under these conditions, the *k*_cat_ values for steady-state ligation of blunt-end, 3′-DSB, and 5′-DSB substrates were all within 2-fold of each other (Table 1). To determine the observed rate constants of sequential ligation events by LIG3, reaction conditions were optimized for detection of the first and second ligation products (Figure 5B and Supplementary Figure S3). Pre-steady-state experiments were performed such that substrate (40 nM) was in 2-fold excess over LIG3 (20 nM) to prevent competitive inhibition of LIG3 by excess LIG3 molecules binding to the DNA ends. The observed rate constants for the first and second ligation events were determined by fitting the reaction time courses to a two-step irreversible model using the kinetics simulation software Berkeley Madonna (Figure 5C) (7,8). For the first ligation event, EJ, the rate constants were within 2-fold of each other for each of the different substrates (Table 1). After the first ligation event LIG3 must undergo conformational changes to release AMP, bind ATP, perform enzyme adenylylation, and reposition itself on the opposite DNA strand to allow for SSB ligation (Figure 5D). The observed rate constants attributed to the second ligation event were similar to those of the initial EJ ligation event (Table 1). Conformational changes associated with adenylylation or strand exchange are likely to limit the overall rate of complete EJ, because LIG3-catalyzed ligation of a SSB is extremely rapid (8). Although sequential ligation could, in principle, involve a dissociation event followed by preferential rebinding of the nicked product, the slow steady-state ligation kinetics suggests the possibility that LIG3 remains bound to the newly ligated DSB to perform sequential ligation events (Figure 5D).

**Table 1.** Rate constants for sequential ligation by LIG3β^a^

*k*_obs_ (min^−1^)	Blunt-end	3′-DSB	5′-DSB
First ligation	3.2 ± 0.2	1.6 ± 0.5	2.6 ± 0.3
Second ligation	4.9 ± 0.3	2.3 ± 0.2	2.4 ± 0.1
Steady-state^b^	0.058 ± 0.012	0.13 ± 0.01	0.11 ± 0.01

^a^Observed single-turnover rate constants for reactions of 20 nM LIG3β and 40 nM EJ substrates in the presence of 1 mM free Mg^2+^ and 10% PEG (Figure 5C).

^b^Averaged *k*_cat_ values for reactions of 1 and 2 nM LIG3β with 20 and 40 nM EJ substrates, respectively. Reactions contained 1 mM free Mg^2+^ and 10% PEG (Figure 5A).

**Figure 6.**
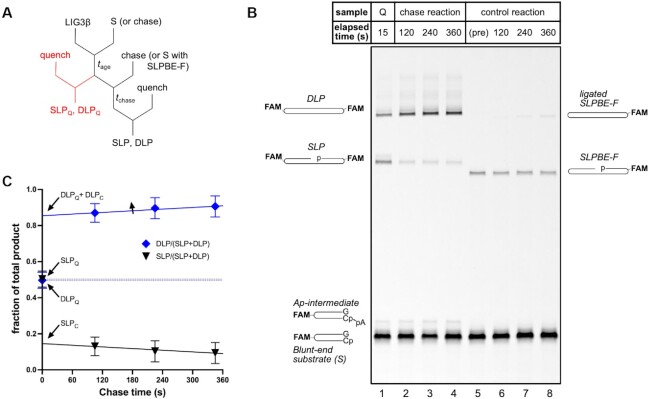
Pulse-chase experiment to measure partitioning of singly ligated product (SLP) between a second ligation event to form DLP and dissociation during LIG3β-catalyzed EJ in 10% (w/v) PEG. (**A**) Schematic of pulse-chase assay. 20 nM LIG3β was mixed with 40 nM blunt-end substrate (S), and after 15 s of aging (*t*_age_) the reaction was either quenched immediately (red pathway) or chased with excess unlabeled SLPBE before quenching (*t*_chase_). Chase was added first in control reactions, as shown in parentheses. (**B**) DNA denaturing gel showing representative samples for the 15 s quench (Q, lane 1), chase reactions (lanes 2–4), and reverse chase control reactions (lanes 5–8). Lane 5 is a pre-addition sample of the mixture of blunt-end substrate and SLPBE-F added as the chase in the reverse chase control reaction. (**C**) Product fraction of DLP (blue diamonds) and SLP (black triangles) immediately before and during the chase period. The total amount of DLP includes the amount that was present after the 15 s aging period (DLP_Q_), the amount that results from sequential ligation (DLP_C_), and a small amount that forms during the chase period from released SLP that slowly rebinds; linear regression was used to determine DLP_Q_+ DLP_C_ and SLP_C_ by extrapolating to zero chase time. Data points (mean ± S.D.) include nine replicates of the 15 s quench (chase time = 0 s) and three replicates of the chase reaction.

To explicitly test the model that a single LIG3 can remain associated with the newly ligated DSB, a pulse-chase experiment to measure partitioning of the singly ligated product between a second ligation event and dissociation from LIG3 was performed (Figure 6). When a pre-steady-state reaction of 20 nM LIG3 and 40 nM of the blunt-end hairpin substrate was quenched after 15 s, a 1:1 product ratio of doubly ligated product (DLP) and singly ligated product (SLP) was present (Figure 6B, lane 1). When the quench was replaced with an excess of an unlabeled, synthetic version of the singly ligated product (SLPBE) to trap any LIG3 that had dissociated, DLP nevertheless increased during the chase period (Figure 6B, C), showing that a substantial portion of the LIG3•SLP intermediate is committed to complete ligation. The 2.5:1 product ratio of DLP_C_ and SLP_C_ in the presence of chase (extrapolated to zero chase time) confirms that sequential ligation of the blunt-end substrate by LIG3 occurs under these conditions and corresponds to an efficiency of ∼0.7 (Equation 7).

The finding that LIG3 remains associated with the nicked DNA intermediate 70% of the time suggests that dissociation from the ligated DNA is at least partially rate-determining for the overall DSB ligation reaction. This finding also demonstrates that LIG3 can adopt the ATP-reactive conformation while associated with DNA. Furthermore, LIG3 must microscopically dissociate and reassociate on the opposite strand to reposition the active site for catalysis. Although it is not clear in the context of a DSB why it would be advantageous to complete both ligation events prior to dissociation, this physical ability is important for the search to find SSBs and DSBs, as an initial binding encounter may not have the proper polarity to engage the catalytic machinery. The ability to perform a processive search is a hallmark of DNA-modifying enzymes that must locate rare sites within the genome. Studies of DNA glycosylases and DNA polymerases show that searching encompasses both strands of the duplex and can bypass other DNA-bound proteins (32–36).

### Conclusions

In support of the recent evidence that LIG1 is capable of performing EJ reactions required for CSR and sister chromatid telomere fusions (21–23), we demonstrate that purified human LIG1 can indeed ligate DNA DSBs with ends that are either blunt or possess short 3′- or 5′-overhangs. At physiological Mg^2+^ levels, the catalytic efficiency of LIG1-catalyzed EJ is reduced relative to the efficient EJ catalyzed by LIG3. However, the catalytic efficiency of LIG1 can be increased 10–100-fold by the addition of PEG, which acts as a molecular crowding agent to mimic the crowded cellular environment. The relatively weak affinity of LIG1 for DSB substrates results in substantial accumulation of abortive EJ species at physiological Mg^2+^ concentration. Although abortive ligation by LIG1 is somewhat reduced upon addition of PEG, it remains a unique feature of LIG1 engagement of DSBs. This is reminiscent of the role abortive ligation plays in enhancing the fidelity of LIG1 by rejecting improperly paired substrates during SSB ligation (37). Abortive ligation of DSB substrates is completely suppressed in the presence of artificially high Mg^2+^ concentrations, which raises the possibility that other *in vivo* factors enhance the efficiency of LIG1 over what we have observed *in vitro*. Under all conditions tested, LIG3 shows higher affinity for DSBs than LIG1, and LIG3 does not generate detectable abortive EJ species. The unique ZnF DNA binding domain that is absent in LIG1 may contribute to the higher DNA affinity of LIG3 and the lack of stimulation by PEG. The transient kinetic analysis for LIG3-catalyzed EJ demonstrates that the tight DNA binding of this enzyme allows it to sequentially ligate both strands of a DSB. The ability of LIG3 to perform sequential ligation events would be beneficial for the faithful ligation of DSBs with compatible ends. However, many physiological breaks contain only a single ligatable end, and in these cases additional processing steps are required to repair the damaged end.

The efficient stand-alone EJ catalyzed by monomeric LIG1 and LIG3 is mechanistically distinct from the classical nonhomologous EJ catalyzed by the LIG4-containing multiprotein complex, recently described in molecular detail (38,39). Whereas LIG1 and LIG3 rely on collision between ligatable DNA ends, the LIG4-containing complex loosely bridges both ends, which allows end-processing and ligation activities to be coupled (38–40). Kinetic analysis of LIG1- and LIG3-catalyzed alternative nonhomologous EJ sheds light on the similarities and differences between the two ligases that will guide future studies of their redundancy and specialized physiological roles in DNA metabolism.

## DATA AVAILABILITY

The data that support the findings of this study are available from the corresponding author.

## Supplementary Material

gkac1263_Supplemental_File
