# Supplementary material for: Human DNA ligases I and III have stand-alone end-joining capability, but differ in ligation efficiency and specificity
Source: Nucleic Acids Res. 2023 Jan 10;51(2):796–805. doi: 10.1093/nar/gkac1263 (PMC9881130; doi:10.1093/nar/gkac1263)
Supplement: gkac1263_Supplemental_File [file gkac1263_supplemental_file.pdf]

Supplementary Data for

Human DNA ligases I and III have stand-alone end-joining capability, but differ in ligation efficiency and specificity

Justin R. McNally, Amanda M. Ames, Suzanne J. Admiraal and Patrick J. O'Brien*

Department of Biological Chemistry, Michigan Medicine, University of Michigan, Ann Arbor, Michigan, 48109, USA

* To whom correspondence should be addressed. Tel: +1 734 647 5821; Fax: +1 734 764 4581; Email: pjobrien@umich.edu

This PDF file includes:

Supplementary Methods

Figure S1. SDS-PAGE gel of purified proteins
Figure S2. Verification of EJ reaction species
Figure S3. Gel-based analysis of sequential ligation events by LIG3β
Figure S4. Dependence of EJ rate on DNA substrate length for LIG1 and LIG3β
Figure S5. Catalytic efficiencies of EJ by LIG1 and LIG3β at saturating Mg^{2+}
Figure S6. Catalytic efficiencies of EJ by LIG1 and LIG3β at physiological Mg^{2+}
Figure S7. Abortive ligation in LIG1 EJ reactions
Figure S8. Abortive ligation by LIG1 occurs independently of DNA concentration.
Figure S9. Catalytic efficiencies of EJ by LIG1 in the presence of 10% PEG

Table S1. Oligonucleotides used to measure and characterize DNA ligation

SUPPLEMENTARY METHODS

Native gel electrophoresis

Oligonucleotides were annealed in annealing buffer (10 mM NaMES, pH 6.5, 50 mM NaCl) by heating the solution to 95 °C for 5 minutes and then cooling to 4 °C at a rate of 12 °C/min. Annealed oligonucleotides were subsequently diluted into standard reaction buffer (pH 7.5) at 150 mM ionic strength. Samples were run on 10% native PAGE (19:1 acrylamide:bisacrylamide, 1×TBE) at constant voltage of 10 V/cm. Fluorescein-labeled DNA was visualized as described for denaturing gels.

Nick ligation of SLPBE-F oligonucleotide

SLPBE-F is a synthetic version of the singly ligated product of EJ of the blunt-end hairpin substrate, and it has one fluorescein label (Table S1). The ligated form of SLPBE-F, which corresponds to the doubly ligated product of EJ, was generated using LIG3β. The nick ligation reaction contained 4 nM LIG3β, 40 nM SLPBE-F, 1 mM ATP, and 2 mM $Mg(OAc)_2$ in standard reaction buffer and was stopped after 10 min at 37 °C with 1.2×quench solution (90% formamide, 50 mM EDTA, 0.006% bromophenol blue 0.006% xylene cyanol).

Exonuclease III treatment of an EJ reaction mixture

An EJ reaction mixture containing blunt-end substrate and its singly and doubly ligated products was generated by incubating 20 nM LIG3β, 40 nM blunt-end substrate (80 nM ends), 1 mM ATP, and 2 mM $Mg(OAc)_2$ in standard reaction buffer with 10% (w/v) PEG for 1 min at room temperature (21 °C). The ligase reaction was stopped by placing the reaction tube at 95 °C for 10 min. 5 uL of this mixture was incubated ± Exonuclease III (50 U, NEB) and 1×NEBuffer 1 at 37 °C for 2.5 hrs before quenching with 1.2×quench solution (90% formamide, 50 mM EDTA, 0.006% bromophenol blue 0.006% xylene cyanol) and running samples on a denaturing gel.

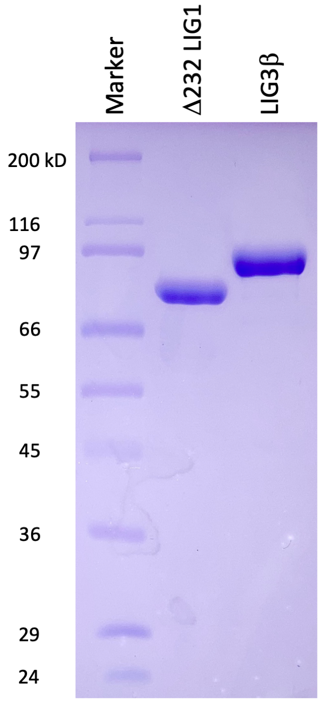

Figure S1. SDS-PAGE gel of purified proteins used in this study: Δ232 LIG1 (76 kD), LIG3β (96 kD).

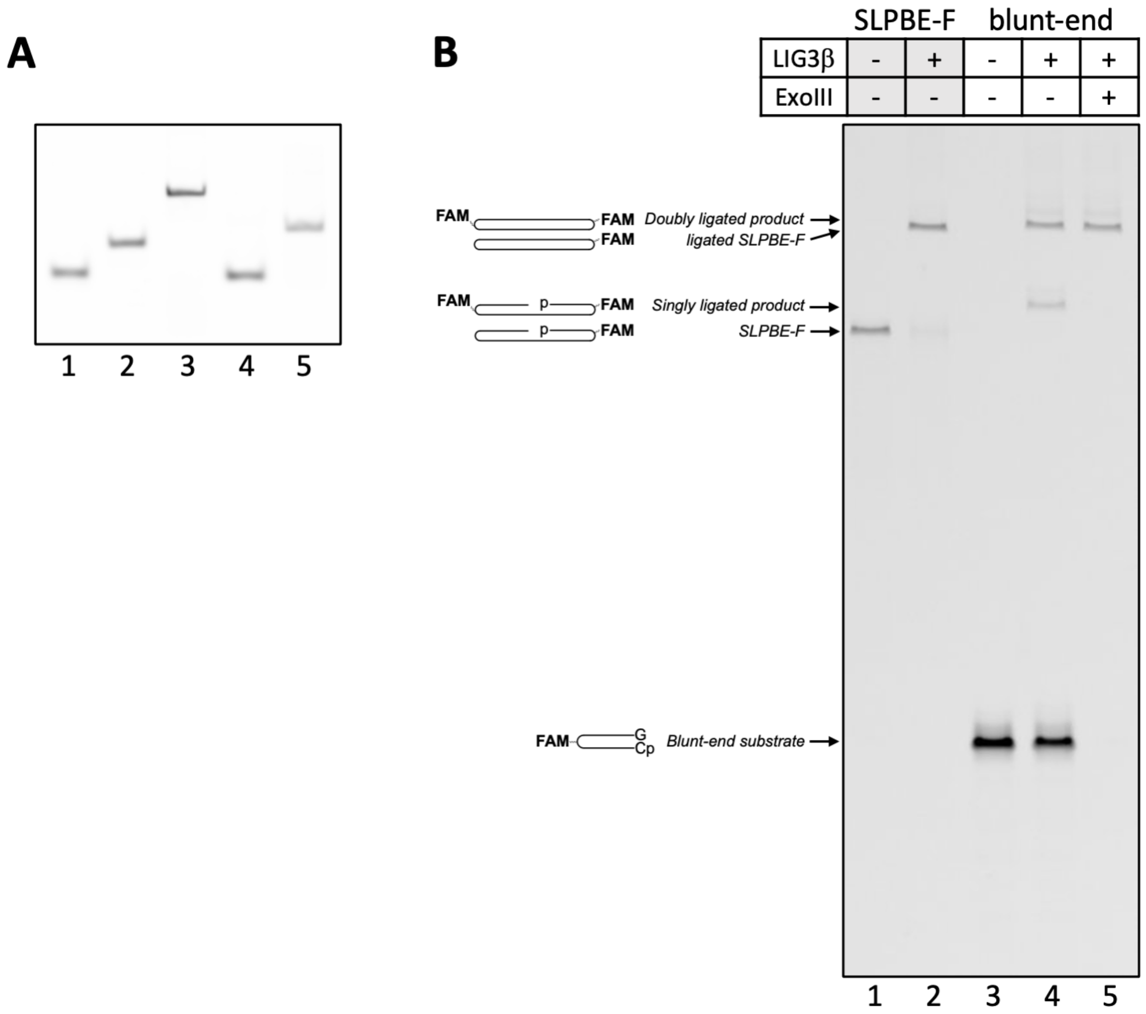

Figure S2. Verification of EJ reaction species. (A) Native gel electrophoresis of the annealed blunt-end substrate and a synthetic version of its singly ligated product (SLPBE-F) in standard reaction buffer. DNA duplexes of 17, 25, and 49 bp were run as size markers in lanes 1–3, respectively. The 31 nt blunt-end substrate (lane 4) migrates at the ~15 bp size that is expected if it is annealed as a hairpin with a 14 bp duplex, rather than as a homodimer that would migrate at twice this size. Likewise, the 62 nt SLPBE-F (lane 5) migrates at the ~30 bp size that is expected if it is annealed as a singly nicked DNA dumbbell. (B) The two products of a reaction of LIG3β with the blunt-end substrate (lane 4) run similarly to a synthetic version of the singly ligated product (SLPBE-F, lane 1) and the ligated product of a reaction of LIG3β with SLPBE-F (lane 2) on a denaturing gel. This identifies the lane 4 products as singly ligated and doubly ligated species; the small mobility differences between SLPBE-F and its ligated product and the corresponding blunt-end substrate ligation products are presumably due to different numbers of fluorescein (FAM), as shown schematically. The unligated blunt-end substrate and its singly ligated product are degraded after 2.5 hr treatment of the lane 4 ligation reaction with Exonuclease III (lane 5), which degrades nicked and linear DNA, but the doubly ligated product is resistant to ExoIII, consistent with it being a sealed species, as shown schematically.

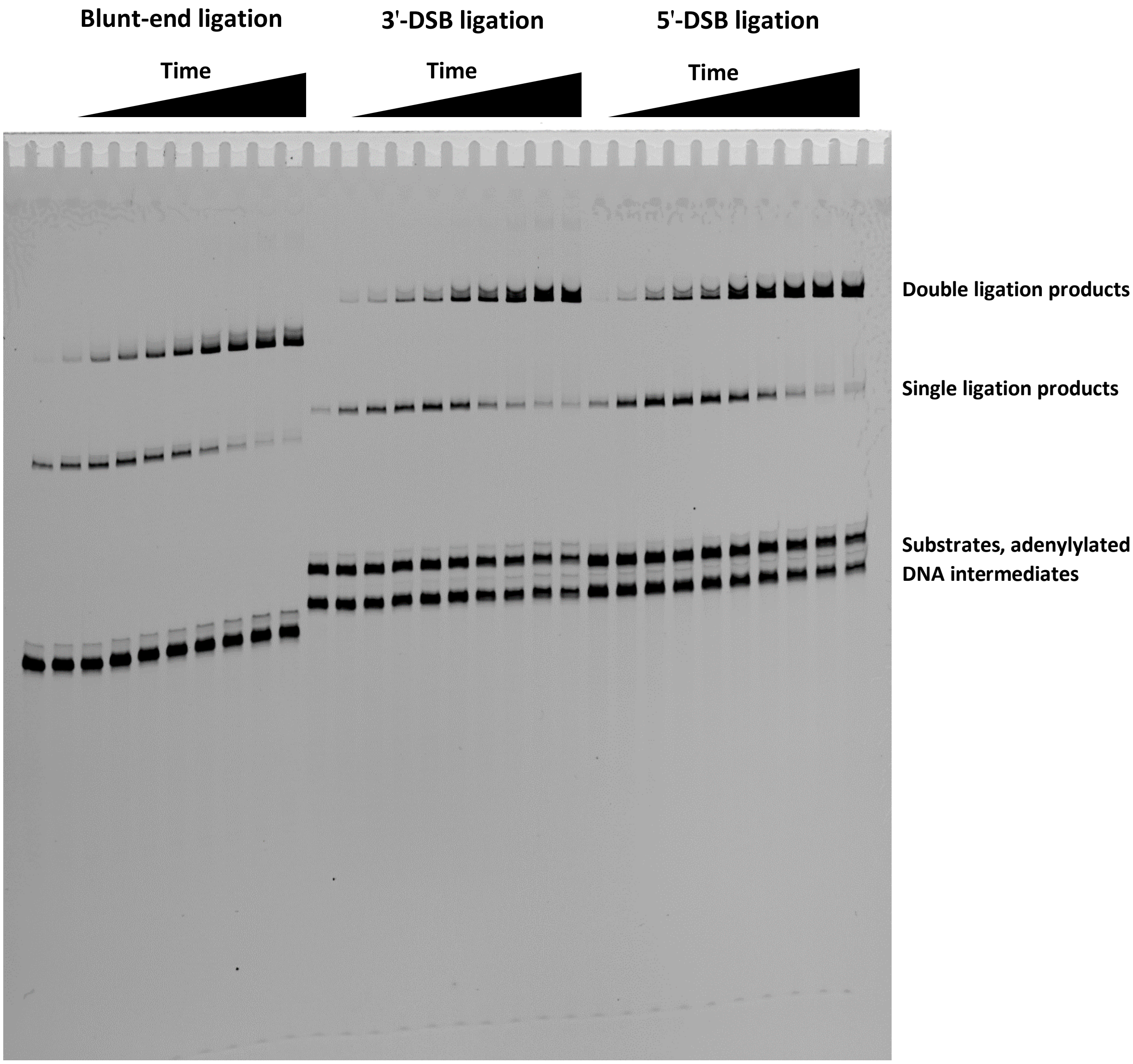

Figure S3. Gel-based analysis of sequential ligation events by LIG3β. Representative DNA denaturing gel showing the formation and consumption of all DNA species observed during single turnover ligation of blunt-end, 3'-DSB, and 5'-DSB substrates by LIG3β. Reactions contained 20 nM LIG3β, 40 nM DNA (80 nM ends), 1 mM ATP, and 2 mM $Mg(OAc)_2$ in standard reaction buffer with 10% (w/v) PEG. Data were acquired from 3–500 s.

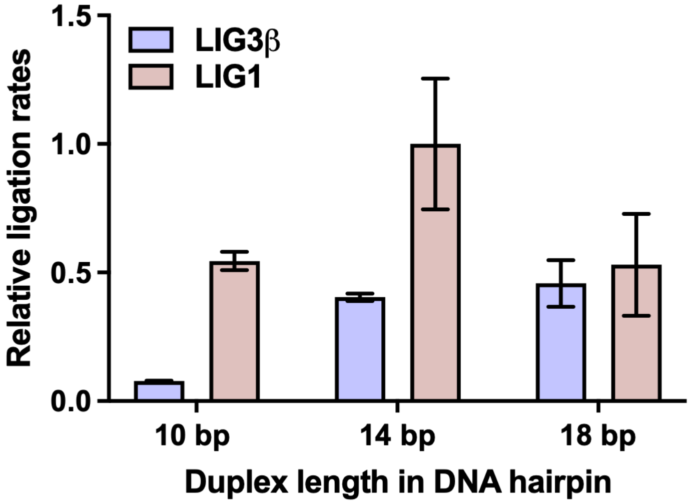

Figure S4. Dependence of EJ rate on DNA substrate length for LIG1 and LIG3β. LIG1-catalyzed EJ rates were within 2-fold of one another for the three blunt-end DNA hairpin substrates tested. LIG3β-catalyzed EJ rates were similar for blunt-end substrates containing 14 bp or 18 bp duplexes, but the EJ rate for the substrate containing a 10 bp duplex was ~5-fold slower. Reaction rates were normalized to the LIG1-catalyzed EJ rate for the substrate with a 14 bp duplex. Reactions contained 5 nM LIG1 or LIG3β, 300 nM DNA substrate (600 nM ligatable ends), and 20 mM Mg^{2+} in standard reaction buffer with ionic strength maintained at 150 mM using NaCl. Oligonucleotide DNA sequences are shown in Table S1. Reactions were performed in duplicate (mean ± S.D.).

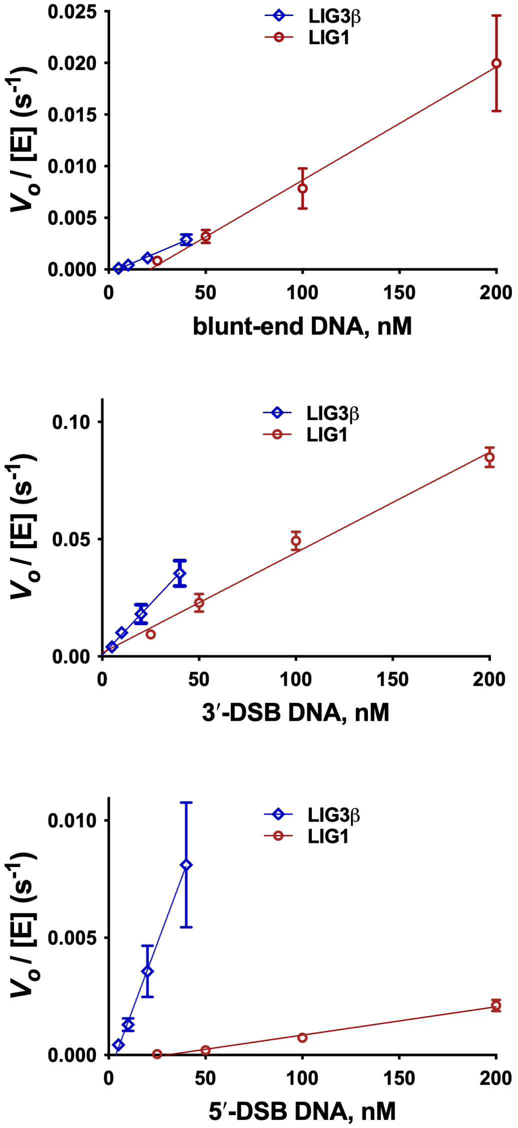

Figure S5. Catalytic efficiencies of EJ by LIG1 and LIG3β at saturating Mg^{2+}. DNA substrate concentration dependence was measured using 5 nM LIG1 and 25–200 nM DNA (red), or 1 nM LIG3β and 5–40 nM DNA (blue). All reactions contained 1 mM ATP and 20 mM $Mg(OAc)_2$ (18 mM free Mg^{2+}, see *Materials and Methods*). Both single and double ligation products were quantified to determine the rate of each EJ reaction. Catalytic efficiency was calculated using linear regression of each DNA concentration dependence. Experiments were conducted in at least triplicate (mean ± S.D.).

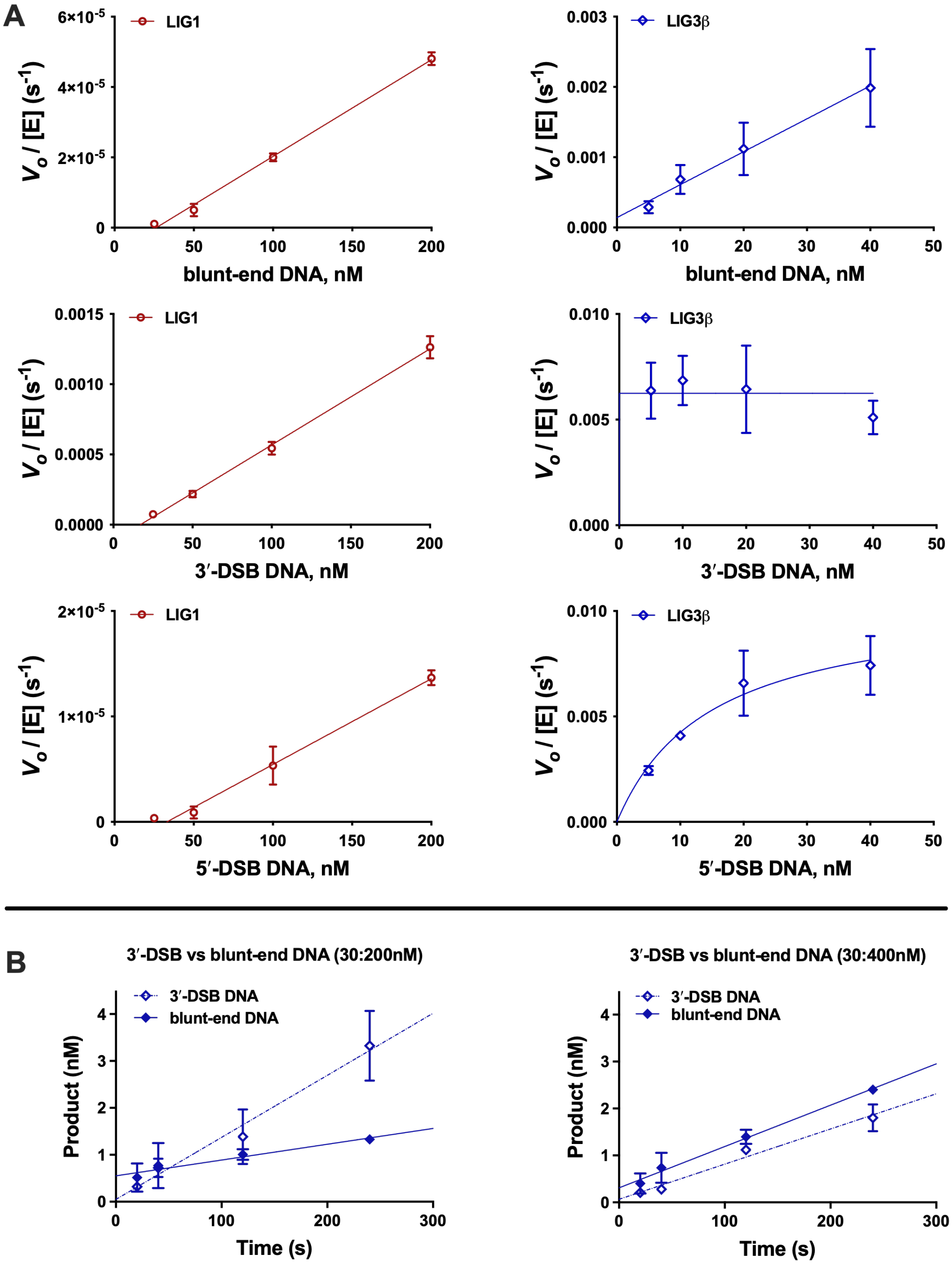

Figure S6. Catalytic efficiencies of EJ by LIG1 and LIG3β at physiological Mg^{2+}. (A) DNA substrate concentration dependence was measured using 5 nM LIG1 and 25–200 nM DNA (red), or 1 nM LIG3β and 5–40 nM DNA (blue). All reactions contained 1 mM ATP and 2 mM $Mg(OAc)_2$ (1 mM free Mg^{2+}, see *Materials and Methods*). Both single and double ligation products were quantified to determine the rate of each EJ reaction. Catalytic efficiency was

calculated using linear regression of the DNA concentration dependences for LIG1-catalyzed EJ and for LIG3β-catalyzed EJ of the blunt-end DNA. Alternative analysis was required to determine the catalytic efficiency of LIG3β with 5'-DSB and 3'-DSB DNA, due to saturation at low DNA concentrations. Rate data for LIG3β-catalyzed EJ of the 5'-DSB substrate was fit to a Michaelis-Menten saturation curve to obtain k_{cat} and K_M values. (B) The catalytic efficiency for LIG3β-catalyzed EJ of the 3'-DSB substrate was determined by direct competition (3'-DSB vs blunt-end; Eq. 2). Direct competition reactions contained 4 nM LIG3β, 2 mM $Mg(OAc)_2$, and 1 mM ATP in standard reaction buffer with 30:200 or 30:400 nM of 3'-DSB:blunt-end DNA, respectively. All experiments were conducted in at least triplicate (mean ± S.D.).

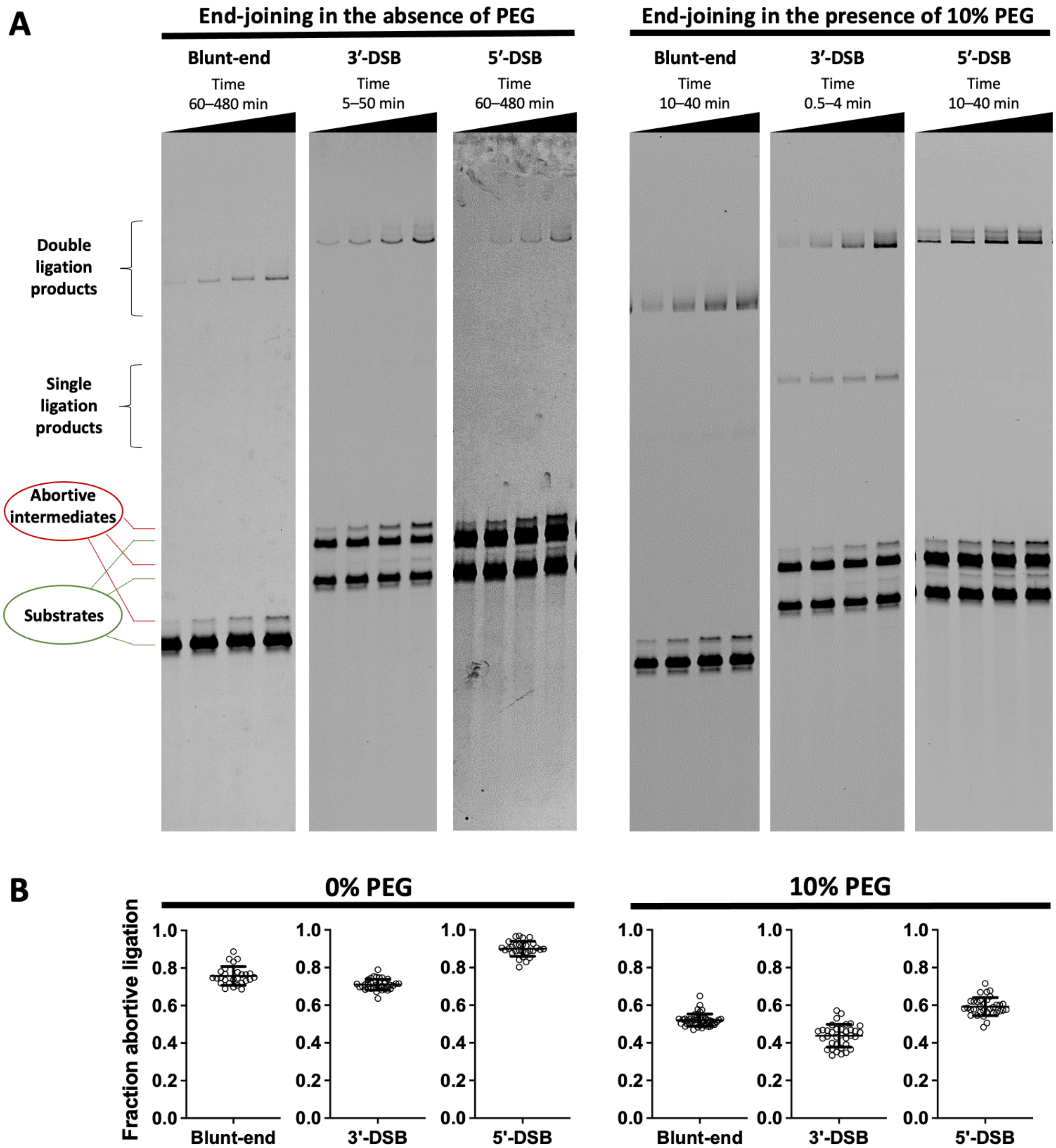

Figure S7. Abortive ligation in LIG1 EJ reactions. (A) Representative DNA denaturing gels illustrate the accumulation of abortive intermediates and ligated products during LIG1-catalyzed EJ in the absence (*left*) and presence (*right*) of 10% (w/v) PEG at 1 mM free Mg^{2+}. Reaction rates increase and fraction abortive ligation decreases in the presence of 10% PEG. (B) Quantification of LIG1-catalyzed abortive ligation (Eq. 5) in the absence (*left*) and presence (*right*) of 10% PEG. Abortive ligation is reduced by 20–30% in the presence of 10% PEG.

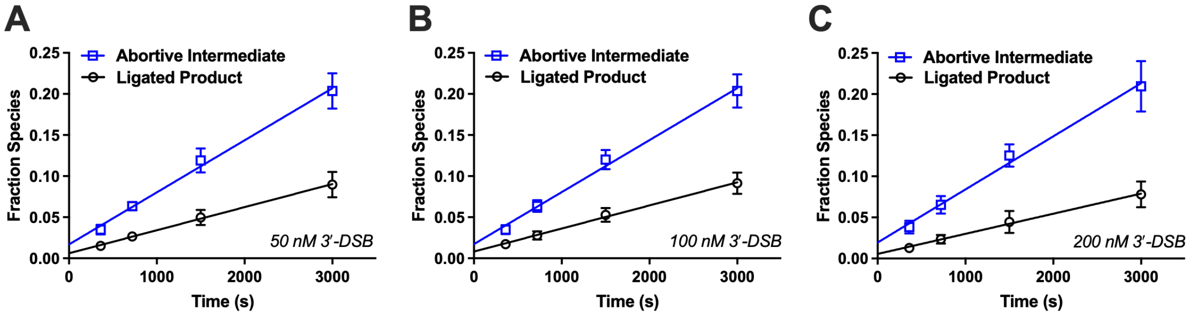

Figure S8. Abortive ligation by LIG1 occurs independently of DNA concentration. At 50 nM (A), 100 nM (B), and 200 nM 3'-DSB (C), LIG1 dissociation from an adenylylated DNA molecule is independent of DNA concentration and substrate/product ratio under steady-state conditions. Reactions contained 5 nM LIG1, 50–200 nM DNA, 2 mM $Mg(OAc)_2$, and 1 mM ATP in reaction buffer. Fig. 3C shows the fraction of all adenylyl transfer events that result in abortive ligation (~70%) at each time point shown above. Reactions were performed in triplicate (mean ± S.D.).

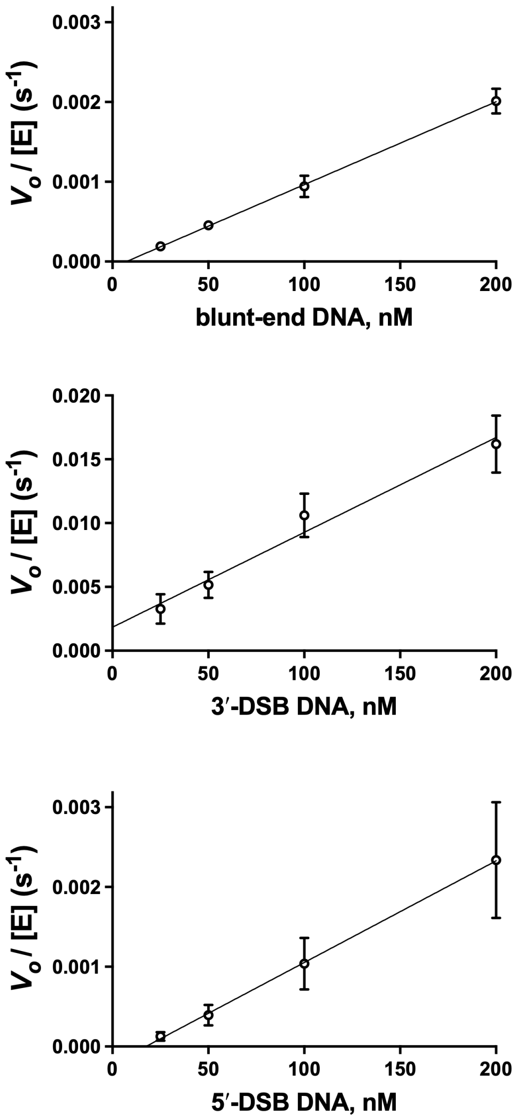

Figure S9. Catalytic efficiencies of EJ by LIG1 in the presence of 10% PEG. DNA substrate concentration dependence was measured using 5 nM LIG1, 25–200 nM DNA, 1 mM ATP, 2 mM $Mg(OAc)_2$, and 10% (w/v) PEG in reaction buffer. Both single and double ligation products were quantified to determine the rate of each EJ reaction. Catalytic efficiency was calculated using linear regression of each DNA concentration dependence. Experiments were conducted in triplicate (mean ± S.D.).

Table S1. Oligonucleotides used to measure and characterize DNA ligation

SSB DNA ligation substrate[a]

up13OH	5'-GTGCTGATGCGTC
P15FAM	5'-pGTCGGACTGATTCGG-**FAM**
Temp28	5'-CCGAATCAGTCCGACGACGCATCAGCAC

DSB DNA ligation substrates[b]

3'-DSB 14 bp duplex	5'-pCATAGAATACTTACG**F**AGTAAGTATTCTATGCAT
3'-DSB 16 bp duplex	5'-pAGAATACTTATTAATCG**F**AGATTAATAAGTATTCTATG
5'-DSB 14 bp duplex	5'-pTACCATAGAATACTTACG**F**AGTAAGTATTCTATG
5'-DSB 16 bp duplex	5'-pGTAAGAATACTTATTAATCG**F**AGATTAATAAGTATTCT
Blunt-end 10 bp duplex	5'-pGAATATTTACG**F**AGTAAATATTC
Blunt-end 14 bp duplex	5'-pCATAGAATATTTACG**F**AGTAAATATTCTATG
Blunt-end 18 bp duplex	5'-pCATAGAATATTTATTAACG**F**AGTTAATAAATATTCTATG

Synthetic versions of DSB DNA singly ligated products[c]

SLPBE-F	5'-pCATAGAATATTTACG**F**AGTAAATATTCTATGCATAGAATATTTACGTAGTAAATATTCTATG
SLPBE	5'-pCATAGAATATTTACGTAGTAAATATTCTATGCATAGAATATTTACGTAGTAAATATTCTATG

[a]The SSB DNA ligation substrate was used to determine active LIG1 and LIG3β concentrations. After annealing, the three oligonucleotides form a duplex DNA molecule with a centrally located nick. The downstream oligonucleotide is 5'-phosphorylated (5'-p) and contains a 3'-fluorescein (-FAM). [b]DSB DNA ligation substrates were used to compare the efficiencies of EJ by LIG1 and LIG3β. Each oligonucleotide was 5'-phosphorylated (5'-p) and contained a dT-fluorescein (F) within a trinucleotide GNA loop, where N is the dT-fluorescein. The three nucleotide overhangs of complementary sequence for paired oligonucleotides comprising the 3'-DSB and 5'-DSB substrates are underlined. [c]Synthetic versions of DSB DNA singly ligated products were 5'-phosphorylated (5'-p). The fluorescently labeled version, SLPBE-F, contained a dT-fluorescein (F) within the trinucleotide GNA loop nearest the 5'-end, where N is the dT-fluorescein.
